# Accuracy and repeatability of the COSMED® Q-NRG max mobile metabolic system

**DOI:** 10.1371/journal.pone.0319394

**Published:** 2025-03-20

**Authors:** Lavinia Falcioni, Laura Guidetti, Carlo Baldari, Andrey Sanko Posada, Chris Wing, Luke Dover, Marco Meucci

**Affiliations:** 1 Department of Public Health and Exercise Science, Appalachian State University, Boone, North Carolina, United States of America; 2 Department of Humanities, Movement, and Education Sciences, University “Niccolò Cusano”, Rome, Italy; 3 Psychology Department, eCampus University, Novedrate, Como, Italy; University of Mississippi, UNITED STATES OF AMERICA

## Abstract

**Purpose:**

To investigate the accuracy and repeatability of the Q-NRG Max® metabolic system against a VacuMed metabolic simulator using a wide range of metabolic rates.

**Methods:**

Sixteen metabolic rates (oxygen consumption 0.9–6 L/min), with different combinations of minute ventilation, oxygen consumption, and carbon dioxide production, were measured for 5 minutes, two times by a single Q-NRG Max® unit over the course of one week. Recordings were performed early in the morning, by the same trained technician, in a ventilated laboratory under the same atmospheric conditions. Accuracy was assessed by ordinary least products (OLP) regression analysis, Bland-Altman plots, intraclass correlation coefficients (ICC), mean percentage differences, technical errors (TE) and minimum detectable change (MDC) for all three variables. This analysis was performed using 10 metabolic rates (oxygen consumption 0.9–4 L/min) and 16 metabolic rates (oxygen consumption 0.9–6 L/min) to allow comparisons with previous research. Intra-device repeatability was performed by absolute percentage differences between measurements (MAPE), ICC, TE, and MDC for the same variables. Repeatability was investigated using 16 metabolic rates.

**Results:**

High agreement and excellent ICCs (>0.998) were observed for all variables when considering both 10 and 16 metabolic rates. The mean percentage difference, TE and MDC were 0.87%–1.01%, 0.67%–1.07%, 1.55%–2.49%, respectively for the first 10 metabolic rates, and −0.39%–0.65%, 0.58%–1.63%, 1.35%–3.81%, respectively for the 16 metabolic rates. The intra-device repeatability results showed an excellent ICCs (=1.000), MAPE < 0.5%, TE < 1%, and MDC ≤ 2%.

**Conclusion:**

The Q-NRG Max® is a valid and reliable mobile metabolic system for the measurement of ventilation, oxygen consumption, and carbon dioxide production. Measurements were below the 5% TE and MDC, and 2% MAPE recommended thresholds across a wide range of metabolic rates up to 6 L/min oxygen consumption.

## Introduction

Automated metabolic systems are considered the gold standard for the assessment of aerobic capacity [1,2]. Historically, these systems have been designed to satisfy researchers’ needs to conduct cardiopulmonary exercise testing (CPET) in laboratory and in real-life conditions using stationary and portable systems [2–5]. Although appropriate for research applications, these machines can be complex and expensive for application in the fitness and performance industry [2,6]. Over the last 20 years, mobile devices have shown to be a valid alternative to stationary systems as their compact design, user-friendly characteristics, and more affordable price allow individuals with less skills and smaller budgets to conduct CPET in different environments [7–9].

The Q-NRG Max® (COSMED, Rome, Italy) is a mobile metabolic system that features a micro-dynamic mixing chamber technology, an oxygen (O_2_) and carbon dioxide (CO_2_) analyzer, a rapid calibration process, and an intuitive touchscreen interface. The Q-NRG Max conducts an automatic calibration before each measurement, offers portability due to its compact dimensions (12.2x8.3x10.6 in) and weight (10.3 lb), operates on either battery power or mains electricity, features an integrated 10“LCD touch screen, utilizes a simplified user interface to reduce the need for advanced technical skills, and integrates with ergometers and ANT+ devices to efficiently and accurately conduct CPET while enabling operators to support athletes and fitness enthusiasts. The seamless integration of these characteristics makes this machine the first system of its kind which is priced at less than half the cost of conventional metabolic carts. However, to our knowledge it’s accuracy and repeatability haven’t been validated yet. It is common practice to validate new metabolic systems against a metabolic simulator as they can effectively reproduce a wide variety of metabolic rates in a short time avoiding some of the drawbacks of the gold standard Douglas Bag method [10–13]. Although findings of research studies using metabolic simulators may not translate to real-world scenarios, they are necessary to assess measurement accuracy (technical validity) of an instrument. This is crucial to ensures that the instrument accurately measures what it is intended to measure guaranteeing that the results obtained from the instrument truly reflect the concept being studied rather than extraneous factors. The majority of validation studies have tested different metabolic systems against a range of metabolic rates with VO_2_ values from 0.5 to 4 L/min [11,14–16]. However, trained individuals and professional athletes can achieve maximal oxygen consumption (VO_2max_) up to 6 L/min [17,18]. Due to the potential application of mobile systems in sport and high-performance testing, a technical validation against a wide range of metabolic rates up to super-athletic VO_2_ is necessary [19–21]. This study aims at investigating the accuracy and of the Q-NRG Max® while measuring metabolic rates from 0.9 to 6 L/min of VO_2_.

## Materials and methods

### COSMED Q-NRG Max®

One Q-NRG Max® (COSMED srl, Italy) was used in this study. The system is equipped with a dynamic micro-mixing chamber, a galvanic O_2_ fuel cell, a non-dispersive infrared digital CO_2_ sensor, and an optoelectronic reader with high-performance T3 turbine flowmeter. Prior to each test and following a 30-min equipment warm-up, flowmeter and gas calibrations were performed. The flowmeter calibration was performed using the 3-L VacuMed syringe with a stroke rate of 20–25 stroke per minute. A gas calibration was performed using room air and a high precision reference gas mixture cylinder (16.00% O_2_, 5.00% CO_2_, balance N_2_) followed by a quality control check sampling 16.00% O_2_ and 5.00% CO_2_ from the cylinder and 20.93% O_2_ and 0.04% CO_2_ from the air. All measured values during the quality control were within ±0.03% of reference values. Before each test, the Q-NRG Max® performed an automatic calibration using room air following the machine’s standard procedures.

### VacuMed metabolic simulator

A commercially available metabolic simulator (Model 17057; VacuMed, Ventura, CA) was used to simulate VO_2_, VCO_2_ and VE [12]. This system was upgraded, calibrated and certified (accuracy of ± 1.00% for simulated VO_2_ and VCO_2_ and ±0.5% for simulated stroke volume) by the manufacturer three months prior to this study. The upgrade allowed the motor-drive syringe pump to deliver stroke volumes from 1 to 5 L, in steps of 0.5 L maintaining the manufacturer’s stroke rates of 6 to 80 strokes^.^min^−1^. The digital mass flow control enables titration of a reference gas cylinder (21.00% CO_2_, 79.00% nitrogen) mixture and its consequent mixing with room air [12]. The VO_2_ and VCO_2_ are expressed in STPD, while the simulator system utilizes known mixtures of a dry tank gas and partially humidified room air. Therefore, the VacuMed software automatically corrects simulated volumes, accounting for temperature, barometric pressure, and humidity measured in room air.

### Study design

**Fig 1:**
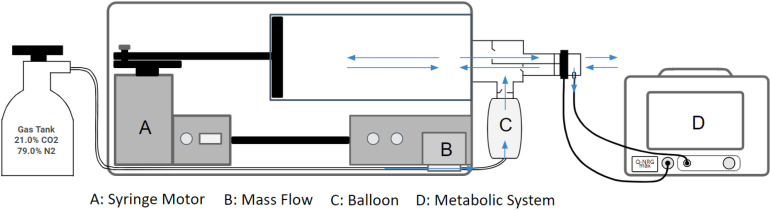
Schematical representation of the experimental design.

The flowmeter and sampling line of the Q-NRG Max® were connected directly to the outlet of the VacuMed metabolic simulator (Fig 1). The Q-NRG Max® uses a patented measurement technology for O_2_ and CO_2_ measurement already adopted in other COSMED portable metabolic carts. Data was provided by the machine every 30 seconds and controlled in real time on the Q-NRG Max® screen during testing. Accuracy and repeatability of the Q-NRG Max® were assessed over 16 metabolic rates (VO_2_ from 0.9 to 6 L/min) lasting 5 minutes each, repeated for two times. Table 1 shows the protocols used for accuracy and repeatability. The selected protocols encompass a broad spectrum of metabolic rates, ranging from a low VO_2_ of 941 mL/min (representing light exercise intensity) to a high VO_2_ of 6063 mL/min (representing very intense exercise typical of athletes), with increments of 100–600 mL/min to simulate a 1–2 MET increase per stage. For each metabolic rate, the corresponding stroke volume and heart rate reflect the values most observed in humans achieving the respective VO_2_. All tests were performed over a one-week period, in the early morning hours, by a single qualified technician, within a well-ventilated laboratory under consistent atmospheric conditions. Protocol stages 1–3 were executed in duplicate on day 1, stages 4–7 were completed in duplicate on day 2, stages 8–10 were repeated twice on day 3, stages 11–13 were duplicated on day 4, and stages 14–16 were performed in duplicate on day 5. Raw data were exported from the Q-NRG Max® and reduced using Excel. Raw data were averaged every 60 seconds and then entered into a spreadsheet for later analysis. To ensure repeatability of results during different days, the tests were performed in an air-conditioned laboratory with consistent atmospheric pressure (690mmHg), ambient temperature (23°C), and relative humidity (25%). Atmospheric conditions were measured by the Q-NRG Max® before and after each test and values were compared to ensure consistency. A fan was placed near the outlet of the metabolic simulator to prevent accumulation of expired air around the Q-NRG Max®. The accuracy and of the Q-NRG Max® were assessed for: VO_2_ (mL/min), VCO_2_ (mL/min) and VE (L/min).

**Table 1:** Test protocol including 16 metabolic rates with VO_2_ from 0.9 to 6 L^.^min^−1^.

	Simulator setting			Simulated values		
Step	Stroke Volume (L)	Stroke Rate (rev/min)	Mass Flow (L/min)	VO_2_ (mL/min)	VCO_2_ (mL/min)	VE (L/min)
1	1.5	15	4.5	941	943	25
2	1.5	25	5.5	1150	1153	42
3	1.5	35	7.5	1568	1572	59
4	2.0	20	6.0	1254	1257	45
5	2.0	30	8.5	1776	1781	67
6	2.0	35	10.5	2194	2200	78
7	2.0	40	13.5	2821	2828	90
8	2.5	35	15.0	3139	3141	97
9	2.5	45	17.0	3558	3560	125
10	2.5	55	19.0	3976	3979	153
11	3.0	50	20.5	4288	4291	167
12	3.0	60	22.5	4706	4709	200
13	3.0	70	25.0	5229	5232	234
14	3.5	45	23.5	4913	4916	176
15	3.5	55	27.0	5645	5648	214
16	3.5	65	29.0	6063	6067	253

Oxygen consumption (VO_2_), carbon dioxide production (VCO_2_), ventilation (VE).

### Statistical analysis

#### Accuracy.

To allow comparisons between the results of this study with previous validation research, measurements were analyzed considering the two ranges of metabolic rates most commonly used in the literature: VO_2_ from 0.9 to 4 mL/min (first 10 metabolic rates), and VO_2_ from 0.9 to 6 mL/min (all 16 metabolic rates). Agreement between the Q-NRG Max® and the VacuMed systems were assessed for VO_2_, VCO_2_ and VE parameters by ordinary least products (OLP) regression analysis, which account for measurement error in both devices [22]. In this analysis the coefficients of determination (R^2^) and slope and intercept with the 95% of confidence intervals (95% CI) were calculated to verify fixed and proportional biases. The hypothesis of proportional and fixed bias was rejected when the 95% CI contained the value 1 for the slope and the 0 for the intercept. Accuracy was quantified as the percentage differences (error) between the Q-NRG Max® and VacuMed simulator $\left[100*\left(\frac{Q-NRGmax\text{®}-VacuMed}{VacuMed}\right)\right]$ and reported as mean and range values, and as the mean absolute percent error (MAPE) between the two values calculated as $MAPE=\left(\frac{1}{n}\right)*\sum \frac{\left|Q-NRGmax\text{®}-VacuMed\right|}{\left|Q-NRGmax\text{®}\right|}*100$. Mean differences were assessed using a paired samples t-test. The intraclass correlation coefficient (ICC) was calculated for criterion accuracy. A single measure, two-way random model, type absolute intra-class correlation coefficient was used to calculate ICCs [23]. Measurement error was expressed as “typical percentage error” (TE), calculated by dividing the standard deviation of the difference score by √ 2 [23], and by “minimum detectable change” (MDC), calculated as $MDC=1.65*\sqrt{2}*TE$. Agreement between the Q-NRG Max® and the VacuMed systems were assessed using Bland-Altman plots and 95% CI between [24]. Analysis was performed using the first 10 metabolic rates (VO_2_ from 0.9 to 4 L/min, low-to-moderate VO_2max_ range commonly used in research studies) and using all 16 metabolic rates (VO_2_ from 0.8 to 6 L/min, low-to-excellent VO_2max_ range including metabolic rates obtained by high-performance athletes).

### Repeatability

Sixteen metabolic rates (from 0.9 to 6 L/min) were reproduced by the metabolic simulator and measured by the Q-NRG Max® twice. The intra-device repeatability of the Q-NRG Max® was evaluated using a single measure, two-way mixed model to calculate ICCs for VO_2_, VCO_2_ and VE [25,26]. Due to the lack of a reference system, the difference between the two trials was quantified as MAPE between measurements of the same Q-NRG Max® as $MAPE=\left(\frac{1}{n}\right)*\sum \frac{\left|actual-forecast\right|}{\left|actual\right|}*100$. The repeatability of the Q-NRG Max® was also assessed using a paired sample t-test for VO_2_, VCO_2_ and VE. Measurement error was expressed in TE% and MDC.

Statistical analyses were performed using the SPSS software (SPSS Inc., IBM, Chicago, IL, USA), with a significance level set at p < 0.05.

## Results

### Accuracy

**Fig 2:**
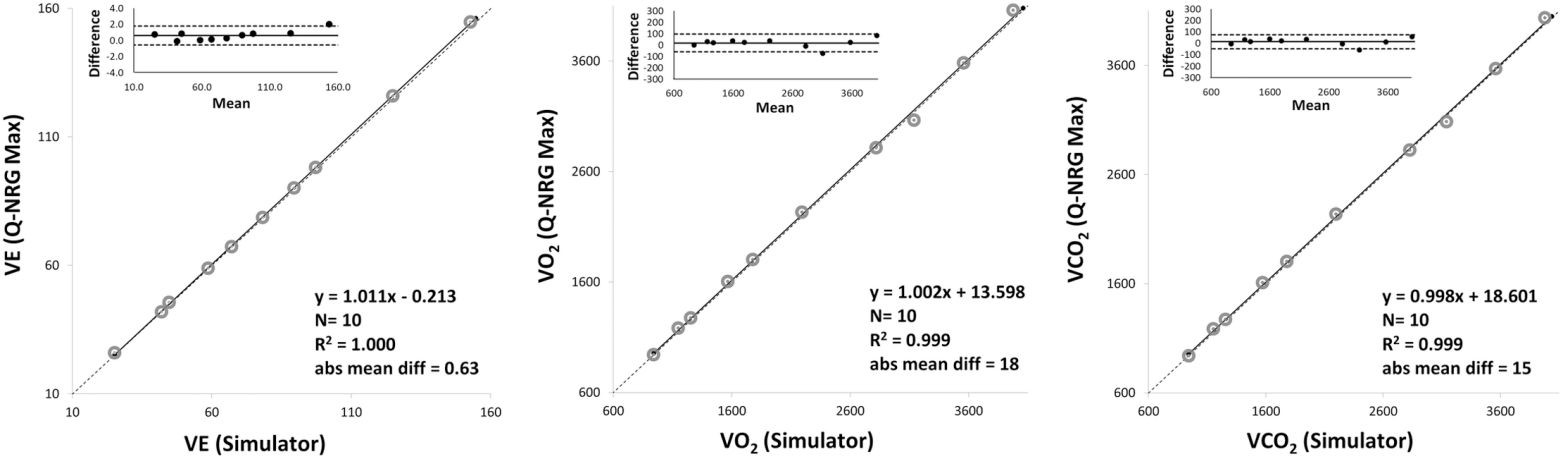
Bland-Altman plots and ordinary least products regression analysis for the first 10 metabolic rates (VO_2_ 0.9–4 L/min).

**Fig 3:**
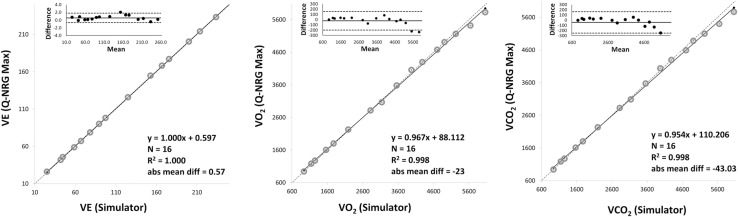
Bland-Altman plots and ordinary least products regression analysis for all the 16 metabolic rates (VO_2_ 0.9–6 L/min).

The agreement between the VacuMed simulator and the Q-NRG Max® is reported in **Table 2**. The results of the OLP regression analysis and of the Bland-Altman plots are shown in **Fig 2** (0.9–4 L/min VO_2_) and **Fig 3** (0.9–6 L/min VO_2_). Each graph reports the OLP regression plot, with the linear regression (solid line), the identity (dashed line), the equation, the determination coefficient (R^2^) and the absolute mean differences, and the Bland-Altman plot (upper-left panel) with the mean percentage difference (solid lines) and the 95% CI (dashed line).

**Table 2:** Agreement between VacuMed simulator and Q-NRG Max.

	Variable	r	Slope (95% CI)	Intercept (95% CI)	Mean diff (%) (min to max)	*p*	ICC (95% CI)	TE (%)	MDC (%)	MAPE (%)
VO_2_ 0.9–4 L/min	VO_2_ (mL/min)	0.999	1.002 (0.973 to 1.032)	13.598 (−52.976 to 78.251)	1.01 ± 1.51 (−2.32 to 2.75)	0.12	0.999 (0.997 to 1.000)	1.07	2.49	1.51
	VCO_2_ (mL/min)	0.999	0.998 (0.976 to 1.022)	18.601 (−33.455 to 69.474)	0.89 ± 1.38 (−1.78 to 2.79)	0.16	1.000 (0.998 to 1.000)	0.97	2.27	1.31
	VE (L/min)	1.000	1.011 (1.002 to 1.020)	−0.213 (−0.896 to 0.461)	0.87 ± 0.94 (−0.27 to 2.89)	0.01*	1.000 (0.997 to 1.000)	0.67	1.55	0.91
VO_2_ 0.9–6 L/min	VO_2_ (mL/min)	0.999	0.967 (0.944 to 0.989)	88.112 (12.285 to 162.192)	0.03 ± 2.07 (−3.98 to 2.75)	0.32	0.999 (0.996 to 0.999)	1.46	3.41	1.60
	VCO_2_ (mL/min)	0.999	0.954 (0.931 to 0.977)	110.206 (33.543 to 185.062)	−0.39 ± 2.31 (−4.98 to 2.79)	0.12	0.998 (0.994 to 0.999)	1.63	3.81	1.81
	VE (L/min)	1.000	1.000 (0.995 to 1.005)	0.597 (−0.008 to 1.200)	0.65 ± 0.82 (−0.27 to 2.89)	0.00*	1.000 (0.999 to 1.000)	0.58	1.35	0.70

Coefficient of correlation (r) slope and intercept of the regression equations, intra-class correlation coefficient (ICC), typical percentage error (TE), minimum detectable change (MDC), and mean absolute percent error (MAPE). Mean difference is reported as mean ±  SD.

*p < 0.05

#### 0.9–4 L/min VO _2_ metabolic rates.

A very strong correlation was observed in VE, VO_2_ and VCO_2_ between the VacuMed and Q-NRG Max® with a R^2^ ranging from 0.999 (VO_2_ and VCO_2_) to 1.000 (VE). No fixed or proportional bias were observed in all variables (slope and intercept include 1 and 0, respectively). The mean percentage difference was 1.01% in VO_2_ (p = 0.1952), 0.89% in VCO_2_ (p = 0.1615), and 0.87% in VE (p < 0.05). ICC values and 95% CI were excellent for all variables (> 0.99). For VE, VO_2_ and VCO_2_ the TE were 0.67%, 1.07% and 0.97%, respectively. For VE, VO_2_ and VCO_2_ the MDC were 1.55%, 2.49% and 2.27%, respectively. Bland-Altman plots show a mean difference of 0.63 L (95% CI of −0.56 and 1.82 L) for VE, 17.73 mL (95% CI of −60.78 and 96.23 mL) for VO_2_, and 15.16 mL (95% CI of −46.43 and 76.74 mL) for VCO_2_.

#### 0.9–6 L/min VO _2_ metabolic rates.

A very strong correlation was observed in VO_2_, VCO_2_ and VE between the VacuMed and Q-NRG Max® with a R^2^ ranging from 0.998 (VO_2_ and VCO_2_) to 1.000 (VE). No fixed or proportional bias were observed in VE (slope and intercept that include 1 and 0, respectively), while proportional bias was observed in VO_2_ and VCO_2_ (intercept values do not include 0). Mean percentage differences were significant for VE (0.65%, p < 0.05) and not significant for VO_2_ (0.03%, p = 0.3184) and VCO2 (−0.39%, p = 0.1246). ICC and 95% CI values were excellent for all variables (> 0.99). For VO_2_, VCO_2_ and VE the TE were 0.58%, 1.46% and 1.63%, respectively. For VO_2_, VCO_2_ and VE the MDC were 1.35%, 3.41% and 3.81%, respectively. Bland-Altman plots show a mean difference of 0.57 L (95% CI of −0.65 and 1.80 L) for VE, −23.23 mL (95% CI of −199.70 mL and 153.24 mL) for VO_2_, and −43.03 mL (95% CI of −250.42 and 164.36 mL) for VCO_2_.

### Repeatability

The results of the repeatability analysis are reported in **Table 3**. The repeatability analysis was performed using all 16 metabolic rates (0.9-6 L/min VO_2_). No significant differences were found in VO_2_, VCO_2_ and VE between trials. For all variables, the MAPE was below 0.5% with the 95% CI values below 1%, the ICC was excellent (= 1.000), and the TE was below 1%. The MDC for VO_2_, VCO_2_ and VE was 1.02%, 1.99% and 2.11%, respectively.

**Table 3:** Results of the Q-NRG Max repeatability test.

Variable	MAPE	p	ICC (95% CI)	TE (%)	MDC (%)
VE (L/min)	0.239 (0.137 to 0.340)	0.87	1.000 (1.000 to 1.000)	0.44	1.02
VO_2_ (mL/min)	0.412 (0.168 to 0.656)	0.18	1.000 (0.999 to 1.000)	0.85	1.99
VCO_2_ (mL/min)	0.396 (0.148 to 0.645)	0.94	1.000 (0.998 to 1.000)	0.90	2.11

Mean absolute percentage difference (MAPE), intra-class correlation coefficient (ICC), typical percentage error (TE), minimum detectable change (MDC) is reported.

## Discussion

The aim of this study was to assess the accuracy and repeatability of the Q-NRG Max® against a metabolic simulator using a wide range of metabolic rates up to super-athletic VO_2_ values. When investigating the accuracy of the machine, to compare results against previous studies, an analysis of the first 10 metabolic rates and one of all 16 metabolic rates was performed.

### Accuracy

#### 0.9–4 L/min VO2 metabolic rates.

The measurements of the Q-NRG Max® showed very high agreement with the simulated values for VO_2_, VCO_2_ and VE when considering metabolic rates with VO_2_ from 0.9 to 4 L/min. All the variables showed high correlation (R^2^ > 0.99), excellent ICC values (> 0.99) and high agreements from the OLP regression and Bland Altman plots. The mean percentage difference in VO_2_ was 1.01% (−2.32 to 2.75) which is lower than ranges reported by other studies conducted on the K5 (−2.55% to 3.52% and −7.27 to 0.03%), Oxycon Pro (5.8% to 10.5%) and COSMED Quark (9% to 12% and 5% to 7%) [11,14,15,27]. The 1.07% TE and the 2.49% MDC were lower than the 5% recommended threshold [1,11,28,29] and lower than the 1.37% TE and 3.79% MDC measured by Guidetti et al. (2018) [11]. The 0.89% (−1.78% to 2.79%) mean percentage difference in VCO_2_ was lower than the 10.5% to 11.7% obtained with the Oxycon pro, the 5% to 7% obtained with the COSMED Quark, and the −6.09% to 2.99% obtained with the COSMED K5 [14,15,27]. The 0.87% (−0.27% to 2.89%) mean percentage difference in VE was lower than the −4.7% to 3.3% reported by VmaxST^tm^, and the 4.2% reported by the COSMED K4 b^2^ [19,20]. The TE and MDC for VCO_2_ (0.97% and 2.27%, respectively) and for VE (0.67% and 1.55%, respectively) were lower than the one reported by Guidetti et al. (2018) (VCO_2_ 1.34% and 3.71%; VE 0.73% and 2.01%, respectively) [11].

#### 0.9–6 L. min-1 VO2 metabolic rates.

A result of high relevance is that the Q-NRG Max® showed to be an accurate system also when considering metabolic rates with VO_2_ from 0.9 to 6 L/min. All variables showed high correlation (R^2^ > 0.99) and excellent ICC values (> 0.99). The mean percentage difference in VO_2_ was −0.03% (−3.98% to 2.75%) which is smaller than the −8.0% (−12.6 to −3.4) mean percentage error obtained with the VmaxSt^tm^ [19] and the 3.6% (up to 7%) obtained with the K4b^2^ [20], and the 7.8% to −3.0% percentage differences obtained with the Cortex MetaMax3B [21]. The greatest difference was observed at the two highest metabolic rates simulating extremely high metabolic conditions (VE and VO_2_ greater than 200 L^.^ min^−1^ and 5.6 L/min, respectively). However, the TE (1.46%) and MDC (3.41%) were lower than the 5% recommended threshold showing acceptable values despite the proportional bias observed by the OLP regression [1,28,29]. This is in line with the results of previous research showing that the accuracy of a metabolic cart may decrease at very high ventilation rates and that the measurement errors are proportional to the magnitude of the simulated values [15,27]. Research indicates that metabolic simulators may lose accuracy at producing very high flow rates when operating near their maximum capacity [30]. This may be due to factors such as increased piston friction potentially altering the piston’s mechanical structure, elevated air temperatures, and the possibility of mechanical linkages occurring within the system. The VCO_2_ showed proportional bias but low TE (1.63%) and MDC (3.81%). The mean percentage difference was −0.39% (−4.98 to 2.79%), which is lower than the −4.6% (−12.0% to 2.8%) for the VmaxST^tm^ [19], the −2.2% for the K4b^2^ [20], and the −0.8% to 10.2% percentage differences for Cortex MetaMax3B [21] studies. The VE measured by the Q-NRG Max® showed a good agreement with the simulated values with no proportional or fixed bias and a mean percentage difference of 0.65% (−0.27% to 2.89%). These results are lower than the 2.5% to 4% reported by Vogler et al. (2010) testing the Cortex MetaMax3B with ventilations up to 240 L/min [21]. The TE (0.58%) and the MDC (1.35%) were lower than the reference values from literature [1].

### Repeatability

The results of the repeatability analysis showed excellent results with ICC values equal to 1.00 in all variables over the 16 simulated metabolic rates. These values are similar to the 0.99–1.00 ICC observed with the COSMED K5 [11,16,27] and higher than the 0.76–0.93 ICC observed with the COSMED Fitmate [31]. The MAPE obtained in this study was below 0.5%, which is lower than the 1% relative error generated from an automated calibration system [10] and the 2% recommended reliability limits [1]. Moreover, this value is lower than what is reported by previous studies indicating a 0.7% to 1.2% MAPE in COSMED K5 and a −0.1% to 2.5% percentage difference in the Cortex Metamax 3B [11,32]. Finally, the Q-NRG Max® reported a 0.44% to 0.90% TE% and 1.02 to 2.11% MDC which is similar to the observed by previous research investigating the COSMED K5 [11,16,27].

### Limitations

A limitation of the present study is the fact that the VacuMed simulator produces a gas mixture at room temperature and humidity [10], only mathematically corrected by the manufacturer’s software. Moreover, the intrinsic accuracy of the metabolic simulator (± 1.00% for VO_2_ and VCO_2_, ± 0.5% for stroke volume) and the lack of biological variability otherwise obtained during real-life measurements in humans affect the generalizability of the results. Therefore, additional validation studies with human subjects are necessary to investigate the validity and reliability of the Q-NRG Max® and confirm its applicability in real-world settings.

## Conclusion

The high agreement, the very high correlation coefficient and the excellent ICC between the Q-NRG Max® and the simulator, together with below recommended threshold percentage difference, TE and MDC make the Q-NRG Max® a valid and reliable mobile system for the measurement of VE, VO_2_, and VCO_2_ up to super-athletic performance.

## Supporting information

S1 File Data. (XLSX)

## References

[1] Hodges LD, Brodie DA, Bromley PD. Validity and reliability of selected commercially available metabolic analyzer systems. Scand J Med Sci Sports. 2005;15(5):271–9. doi: 10.1111/j.1600-0838.2005.00477.x 16181250

[2] Macfarlane D. Automated metabolic gas analysis systems: a review. Sports Med. 2001;31(12):841–61. 11665912 10.2165/00007256-200131120-00002

[3] Meyer T, Davison RCR, Kindermann W. Ambulatory gas exchange measurements--current status and future options. Int J Sports Med. 2005;26(Suppl 1):S19–27. doi: 10.1055/s-2004-830507 15702452

[4] Perez-Suarez I, Martin-Rincon M, Gonzalez-Henriquez JJ, Fezzardi C, Perez-Regalado S, Galvan-Alvarez V, et al. Accuracy and precision of the COSMED K5 portable analyser. Front Physiol. 2018;9:1764. doi: 10.3389/fphys.2018.01764 30622475 PMC6308190

[5] Tsekouras YE, Tambalis KD, Sarras SE, Antoniou AK, Kokkinos P, Sidossis LS. Validity and reliability of the new portable metabolic analyzer PNOE. Front Sports Act Living. 2019;1:24. doi: 10.3389/fspor.2019.00024 33344948 PMC7739780

[6] Macfarlane DJ. Open-circuit respirometry: a historical review of portable gas analysis systems. Eur J Appl Physiol. 2017;117(12):2369–86. doi: 10.1007/s00421-017-3716-8 29043499

[7] Dieli-Conwright CM, Jensky NE, Battaglia GM, McCauley SA, Schroeder ET. Validation of the CardioCoachCO2 for submaximal and maximal metabolic exercise testing. J Strength Cond Res. 2009;23(4):1316–20. doi: 10.1519/JSC.0b013e3181a3c5e8 19528838

[8] Nieman D, Lasasso H, Austin M, Pearce S, McInnis T, Unick J. Validation of Cosmed’s FitMate^TM^ in measuring exercise metabolism. Res Sports Med (Print). 2007 Mar 1;15:67–75. 10.1080/15438620601184380 17365953

[9] Vehrs PR, Keller DM, George JD, Hager RL, Fellingham GW. Monitoring VO2max during fourteen weeks of endurance training using the CardioCoach. J Strength Cond Res. 2007;21(1):62–6. doi: 10.1519/00124278-200702000-00012 17313267

[10] Gore CJ, Catcheside PG, French SN, Bennett JM, Laforgia J. Automated VO2max calibrator for open-circuit indirect calorimetry systems. Med Sci Sports Exerc. 1997;29(8):1095–103. doi: 10.1097/00005768-199708000-00016 9268968

[11] Guidetti L, Meucci M, Bolletta F, Emerenziani GP, Gallotta MC, Baldari C. Validity, reliability and minimum detectable change of COSMED K5 portable gas exchange system in breath-by-breath mode. PLoS One. 2018;13(12):e0209925. doi: 10.1371/journal.pone.0209925 30596748 PMC6312326

[12] Huszczuk A, Whipp BJ, Wasserman K. A respiratory gas exchange simulator for routine calibration in metabolic studies. Eur Respir J. 1990;3(4):465–8. doi: 10.1183/09031936.93.03040465 2114308

[13] Souren T, Rose E, Groepenhoff H. Comparison of two metabolic simulators used for gas exchange verification in cardiopulmonary exercise test carts. Front Physiol. 2021;12:667386. doi: 10.3389/fphys.2021.667386 34149449 PMC8209337

[14] Beijst C, Schep G, Breda E van, Wijn PFF, Pul C van. Accuracy and precision of CPET equipment: a comparison of breath-by-breath and mixing chamber systems. J Med Eng Technol. 2013;37(1):35–42. doi: 10.3109/03091902.2012.733057 23110656

[15] Carter J, Jeukendrup AE. Validity and reliability of three commercially available breath-by-breath respiratory systems. Eur J Appl Physiol. 2002;86(5):435–41. doi: 10.1007/s00421-001-0572-2 11882930

[16] Winkert K, Kamnig R, Kirsten J, Steinacker JM, Treff G. Inter- and intra-unit reliability of the COSMED K5: Implications for multicentric and longitudinal testing. PLoS One. 2020;15(10):e0241079. doi: 10.1371/journal.pone.0241079 33096546 PMC7584194

[17] Alejo LB, Montalvo-Pérez A, Valenzuela PL, Revuelta C, Ozcoidi LM, de la Calle V, et al. Comparative analysis of endurance, strength and body composition indicators in professional, under-23 and junior cyclists. Front Physiol. 2022;13:945552. doi: 10.3389/fphys.2022.945552 35991188 PMC9388719

[18] Faria IE, Faria EW, Roberts S, Yoshimura D. Comparison of physical and physiological characteristics in elite young and mature cyclists. Res Q Exerc Sport. 1989;60(4):388–95. doi: 10.1080/02701367.1989.10607469 2489869

[19] Prieur F, Castells J, Denis C. A methodology to assess the accuracy of a portable metabolic system (VmaxST). Med Sci Sports Exerc. 2003;35(5):879–85. doi: 10.1249/01.MSS.0000065003.82941.B0 12750601

[20] Rodríguez FA, Keskinen KL, Kusch M, Hoffmann U. Validity of a swimming snorkel for metabolic testing. Int J Sports Med. 2008;29(2):120–8. doi: 10.1055/s-2007-964973 17960507

[21] Vogler AJ, Rice AJ, Gore CJ. Validity and reliability of the Cortex MetaMax3B portable metabolic system. J Sports Sci. 2010;28(7):733–42. doi: 10.1080/02640410903582776 20419553

[22] Ludbrook J. Linear regression analysis for comparing two measurers or methods of measurement: but which regression? Clin Exp Pharmacol Physiol. 2010;37(7):692–9. doi: 10.1111/j.1440-1681.2010.05376.x 20337658

[23] Hopkins WG. Measures of reliability in sports medicine and science. Sports Med. 2000;30(1):1–15. doi: 10.2165/00007256-200030010-00001 10907753

[24] Bland JM, Altman DG. Measuring agreement in method comparison studies. Stat Methods Med Res. 1999;8(2):135–60. doi: 10.1191/096228099673819272 10501650

[25] Koo TK, Li MY. A guideline of selecting and reporting intraclass correlation coefficients for reliability research. J Chiropr Med. 2016;15(2):155–63. doi: 10.1016/j.jcm.2016.02.012 27330520 PMC4913118

[26] McGraw KO, Wong SP. Forming inferences about some intraclass correlation coefficients. Psychol Methods. 1996;1(1):30–46. doi: 10.1037/1082-989x.1.1.30

[27] Winkert K, Kirsten J, Dreyhaupt J, Steinacker JM, Treff G. The COSMED K5 in breath-by-breath and mixing chamber mode at low to high intensities. Med Sci Sports Exerc. 2020;52(5):1153–62. doi: 10.1249/MSS.0000000000002241 31895296

[28] Babineau C, Léger L, Long A, Bosquet L. Variability of maximum oxygen consumption measurement in various metabolic systems. J Strength Condit Res. 1999;13(4):318–24. doi: 10.1519/00124278-199911000-00004

[29] Overstreet BS, Bassett DR Jr, Crouter SE, Rider BC, Parr BB. Portable open-circuit spirometry systems. J Sports Med Phys Fitness. 2017;57(3):227–37. doi: 10.23736/S0022-4707.16.06049-7 26861831

[30] Souren T, Rose E, Groepenhoff H. Comparison of two metabolic simulators used for gas exchange verification in cardiopulmonary exercise test carts. Front Physiol. 2021 June;12. 10.3389/fphys.2021.667386 PMC8209337 34149449

[31] Brisswalter J, Peikriszwili Tartaruga M. Comparison of COSMED’S FitMate (TM) and K4b2 metabolic systems reliability during graded cycling exercise. Scandinavian J Clin Lab Invest. 2014;74(11):722–4. 10.3109/00365513.2014.930711 25375032

[32] Macfarlane DJ, Wong P. Validity, reliability and stability of the portable Cortex Metamax 3B gas analysis system. Eur J Appl Physiol. 2012;112(7):2539–47. doi: 10.1007/s00421-011-2230-7 22075643 PMC3371330

